# Bridging the gap in musculoskeletal ultrasound training for rheumatology trainees: development of a structured training pathway in Northern Ireland

**DOI:** 10.1093/rap/rkag092

**Published:** 2026-07-23

**Authors:** Katarzyna Nowak, Ashley Elliott, Michelle McHenry

**Affiliations:** Department of Rheumatology, Musgrave Park Hospital, Belfast, Northern Ireland; Department of Rheumatology, Musgrave Park Hospital, Belfast, Northern Ireland; Department of Rheumatology, Musgrave Park Hospital, Belfast, Northern Ireland

**Keywords:** musculoskeletal ultrasound, rheumatology training, logbook, structured training pathway

## Abstract

**Objectives:**

Musculoskeletal ultrasound has become an important tool in rheumatology practice. Despite its increasing use in the UK, there are currently no nationally standardised guidelines for rheumatology trainees. Recommendations for ultrasound training and education have been proposed but training opportunities and assessment of competency remains variable. This project aimed to evaluate the current ultrasound training practices among rheumatology trainees in Northern Ireland and to develop a structured approach to training in order to help trainees obtain formal ultrasound qualification.

**Methods:**

A baseline survey was conducted among rheumatology trainees in Northern Ireland to assess current ultrasound practice, supervision, documentation and awareness of certification pathways. The survey also collected suggestions for improving training and competency development.

**Results:**

The survey demonstrated that most trainees perform ultrasound at least a few times a week; however, supervision remains variable. Only one trainee has a structured logbook at present. Based on the findings of the survey, a structured musculoskeletal ultrasound logbook was developed. This aligns with European Federation of Societies for Ultrasound in Medicine and Biology level 1 and EULAR curriculum. It was introduced at an ultrasound teaching day together with a Bingo game that was developed to further support trainee engagement.

**Conclusions:**

This project identified significant variability in ultrasound training practices among rheumatology trainees in Northern Ireland. The introduction of a structured ultrasound logbook, targeted teaching sessions and innovative educational tools aims to standardise training and facilitate progression towards formal certification.

Key messages

- Musculoskeletal ultrasound has become an important tool in everyday rheumatology practice.
- There are currently no nationally standardised guidelines on ultrasound training requirements for rheumatology trainees.
- By introducing an ultrasound logbook we aim to standardise training and help assist trainees in obtaining formal certification.

## Introduction

Over the last ≥20 years, rheumatologists have been utilising bedside musculoskeletal ultrasound to help them with clinical decision-making. The use of ultrasound is growing continuously, especially in inflammatory arthritis, giant cell arteritis and in the field of guided interventions. Currently in the UK there are no national guidelines on ultrasound training. In 2020, Atachia *et al.* [1] published the ‘Recommendations for rheumatology ultrasound training and practice in the UK’. In this article they set the first specific education and practice recommendations for rheumatology ultrasound in the UK. These have been endorsed by the BSR Ultrasound Special Interest Group. In Northern Ireland, a need for standardised training both locally and within national rheumatology training schemes was highlighted as far back as 2006 [2].

The Royal College of Physicians of Edinburgh describes rheumatology as a ‘rapidly progressing speciality’ and in their training program overview they mention that ‘skills in the use of ultrasound are also desirable’ to complement this expansion.

Similarly, the Joint Royal Colleges of Physicians Training Board Rheumatology Curriculum 2022 recognises that ultrasound is ‘increasingly utilised as part of daily practice and many rheumatologists are competent at using musculoskeletal ultrasound’. They do not define what the competence is and ultrasound-guided joint or soft tissue injections are listed as ‘optional’ procedures for trainees.

In contrast to that, the EULAR and ACR have set out training curricula and minimum requirements for different competency levels and have developed a system of practical training and certification of competency.

The European Federation of Societies for Ultrasound in Medicine and Biology (EFSUMB) governs training and practice standards in Europe and has published ‘Minimum training requirements for rheumatologists performing musculoskeletal ultrasound’ in conjunction with the EULAR, which is the representative body for all rheumatology societies in Europe [3]. These recommendations establish the theoretical and practical curriculum for training and achieving competency in ultrasound in rheumatology.

Interestingly, the German Society for Rheumatology updated their curriculum for advanced training in internal medicine and rheumatology in 2022. Their specialised training period in internal medicine and rheumatology extends over 36 months. The curriculum defines that, by the end of third training year, the trainee should independently perform targeted rheumatologic procedures using arthro- and vascular sonography (including duplex sonography for acute diagnosis of vasculitides), as well as ultrasound of the salivary glands [4].

## Methods

The aim of this project is to define the current musculoskeletal ultrasound training practice among rheumatology trainees in Northern Ireland. It also aims to develop a more structured approach to ultrasound training and obtaining formal musculoskeletal ultrasound certification.

We conducted a baseline survey developed by the study authors that was distributed among all rheumatology trainees in Northern Ireland (*n* = 8) to establish current ultrasound teaching practices and identify potential areas for improvement. The full survey questionnaire is included in the supplementary material (Supplementary Table S1). The survey explored the current level of ultrasound use in daily practice, previous attendance at formal ultrasound courses, intentions to complete the EFSUMB level 1 examination before the end of training and suggestions for improving future musculoskeletal ultrasound training.

## Results

Six trainees completed the survey and responses were anonymised. The baseline survey demonstrated that most trainees (83.33%) performed ultrasound at least a few times a week. Levels of supervision varied, with 50% reporting receiving instant feedback approximately once a month. Half of the trainees reported no knowledge as to how to save ultrasound images. Currently, only one trainee was maintaining a logbook of scans, with the majority (83.33%) indicating that they plan to sit for the EFSUMB level 1 musculoskeletal ultrasound examination before completion of training.

## Discussion

Musculoskeletal ultrasound has become an increasingly important tool in modern rheumatology practice. It helps to support the diagnosis and monitor disease activity and is being used for image-guided injections. Despite this growing role, our findings suggest that ultrasound training for rheumatology trainees in Northern Ireland remains variable and largely depends on local opportunities rather than a structured framework. Ultrasound is integrated into routine rheumatology practice, as reported by our trainees. However, without a structured system for documenting scans and demonstrating competency, achieving a formal qualification can be challenging. The absence of nationally mandated training standards in the UK may contribute to this variability. While the Joint Royal Colleges of Physicians Training Board rheumatology speciality training curriculum acknowledges the increasing role of ultrasound in clinical practice, it does not clearly define expected competencies or minimum training requirements. Incorporating ultrasound competency expectations directly into the rheumatology training curricula, following the example of training programs in Germany and the Republic of Ireland, would demonstrate both the feasibility and the value of having clearly structured training pathways.

Based on the survey findings, we identified a need to restructure musculoskeletal ultrasound training in Northern Ireland.

Our project sought to address some of the gaps identified in ultrasound training locally. A structured ultrasound logbook was therefore developed to support trainees in achieving formal ultrasound accreditation during training. Trainees are expected to complete a minimum of 300 scans overall, including at least 25 scans in each joint region: shoulder, elbow, wrist, hand, hip, knee, ankle and foot. Temporal artery scanning is also encouraged, although this component is not currently mandatory.

The logbook provides a framework for documenting scans, storing images, recording feedback from supervising consultants and tracking progress towards competency achievement. Furthermore, it aims to standardise training experiences in Northern Ireland and support trainees preparing for formal certification. The logbook includes the ‘MSK Ultrasound Bingo’ game, and we believe this strategy will be a useful tool to enhance trainee motivation and engagement. The bingo card provides an accessible and enjoyable way for trainees to structure their learning and progressively build their logbook of images.

**Figure 1:**
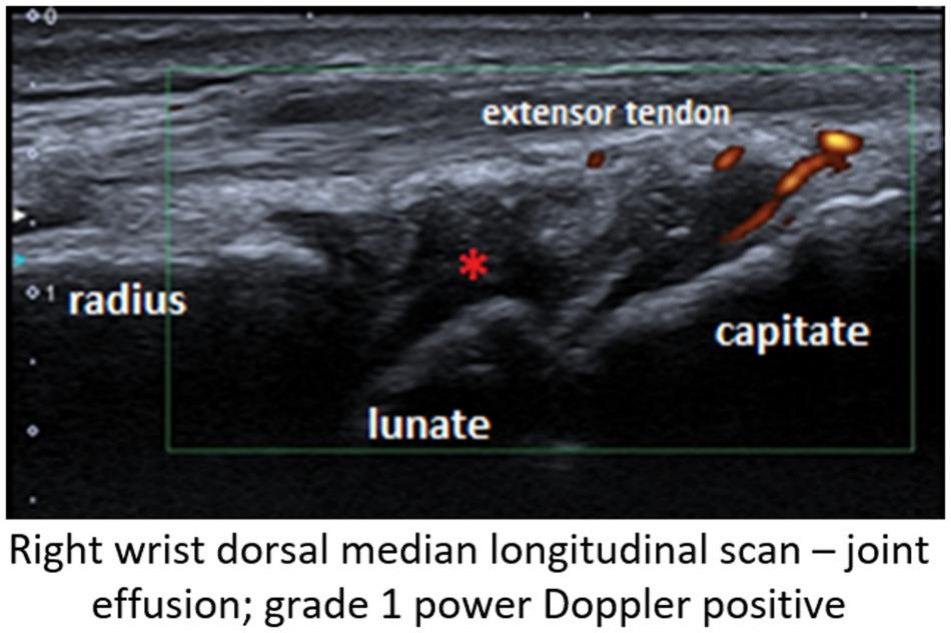
Example image to be included in the ultrasound logbook. The type of scan, important anatomical landmarks and the abnormality should be labelled

The Musculoskeletal Ultrasound Logbook (available in full as Supplementary Table S2) was introduced during an ultrasound teaching day. The logbook was adapted from the format currently used by trainees in the Republic of Ireland and incorporates the EFSUMB level 1 curriculum as a training template, alongside a suggested training schedule. In addition, the logbook provides examples of appropriate image presentation (Fig. 1).

A dedicated rheumatology registrar teaching day was delivered in February 2026. The program focused on practical aspects of ultrasound training, including the use of the logbook, image storage and the structure of the EFSUMB level 1 examination. Teaching also covered ultrasound definitions, scoring systems and scanning techniques for temporal arteries, salivary glands and entheses. The session incorporated both theoretical and practical components.

We aim to continue to deliver those on a regular basis going forward. We will also integrate training with established basic and intermediate courses already offered in musculoskeletal ultrasound in Belfast and Dublin and throughout the UK.

Following from the success of the ‘Rheum Week Bingo’ card developed by Gillispie-Taylor [5], we have created our own ‘MSK Ultrasound Bingo’ card in order to motivate trainees and help to guide and prioritise their learning. Topics for the bingo card were selected from the EFSUMB level 1 curriculum. ‘Bingo’ can be achieved by having five consecutive squares (either horizontally, vertically or diagonally) each signed off by a supervising consultant with accompanying images included in the logbook. The first trainee to report ‘BINGO’ between February 2026 and July 2026 will receive a certificate and a small prize.

Taken together, these interventions represent an important step towards a more structured and supportive musculoskeletal ultrasound training pathway for rheumatology trainees in Northern Ireland. We recognise several limitations of our project, including the relatively small number of rheumatology trainees in Northern Ireland. To minimise the risk of self-reporting bias, the baseline survey was anonymised. The potential for selection bias was also reduced by ensuring all trainees received the survey link and were given sufficient time to respond, with the survey remaining open for 3 weeks. Although this project focuses on a single training region, it highlights broader challenges that are likely shared across many regions within the UK. Another important limitation is the lack of formal evaluation of the educational impact of our interventions to date. While we plan to assess uptake of the ‘MSK Ultrasound Bingo’ game, trainee engagement with the logbook and changes in trainee confidence levels, these outcomes have not yet been formally evaluated. Future work will therefore focus on assessing the impact of the logbook, teaching sessions and related educational initiatives on trainee competency development and success in formal ultrasound certification. In the longer term, there may also be a value in developing nationally coordinated musculoskeletal ultrasound training standards aligned with established international frameworks.

## Supplementary Material

rkag092_Supplementary_Data

## Data Availability

The data underlying this article are available in the article and in its online supplementary material.

## References

[1] Atchia I , Brown AK, Chitale S et al Recommendations for rheumatology ultrasound training and practice in the UK. Rheumatology (Oxford) 2021:60:2647–52. doi: 10.1093/rheumatology/keaa656 33167033

[2] Taggart A , Filippucci E, Wright G et al Musculoskeletal ultrasound training in rheumatology: the Belfast experience. Rheumatology (Oxford) 2006;45:102–5. 10.1093/rheumatology/kei162 16263780

[3] Terslev L , Hammer HB, Torp-Pedersen S et al EFSUMB minimum training requirements for rheumatologists performing musculoskeletal ultrasound. Ultraschall Med 2013;34:475–7. doi: 10.1055/s-0033-1335143 23696065

[4] Pfeil A , Krusche M, Vossen D et al Model curriculum of the German Society for Rheumatology for advanced training in the discipline internal medicine and rheumatology. English version. Z Rheumatol 2021;80(Suppl 2):64–7. doi: 10.1007/s00393-021-01080-6 34605981 PMC8752525

[5] Gillispie-Taylor M. Bingo! Gamifying pediatric rheumatology education one square at a time. Perspect Med Educ 2025;14:399–404. https://doi. org/10.5334/pme.1749 10.5334/pme.1749 PMC12493062 41048370

