# Supplementary material for: Bridging the gap in musculoskeletal ultrasound training for rheumatology trainees: development of a structured training pathway in Northern Ireland
Source: Rheumatol Adv Pract. 2026 Jul 23;10(3):rkag092. doi: 10.1093/rap/rkag092 (PMC13451082; doi:10.1093/rap/rkag092)
Supplement: Supplementary file 1 [file rkag092_supplementary_data.docx]

**Supplementary material**

**Supplementary data S1**: Baseline musculoskeletal ultrasound survey questionnaire

1. Currently, how frequently are you using ultrasound in your day-to-day practice?
   a. Every day
   b. A few times a week
   c. About once a week
   d. A few times a month
   e. Once a month
   f. Less than once a month
2. How often do you get to perform ultrasound under supervision? (and receive feedback)
   a. Never – I just always scan on my own
   b. Very rarely – once every few months
   c. Once a month
   d. Weekly
   e. A couple of times per week
3. Have you attended any formal ultrasound teaching?
   a. Local departmental teaching sessions
   b. SpR teaching days
   c. Basic course
   d. Intermediate course
   e. Advanced course
   f. EULAR online US course
   g. Others
4. Do you know how to save images and transfer them onto a computer?
   a. Yes
   b. No
5. Do you currently have an ultrasound logbook?
   a. Yes
   b. No
6. If yes, what logbook are you using to keep images? Free text.
7. Do you plan to sit the EFSUMB Level 1 exam by the end of your training?
   a. Yes
   b. No
8. Do you have any suggestions how ultrasound teaching could be improved for rheumatology trainees in Northern Ireland? Free text.

**Supplementary data S2:** Musculoskeletal Ultrasound Training Logbook

**Musgrave Park Hospital Belfast Ultrasound Special Interest Group**

**Dr Gary Wright**

**Dr Michelle McHenry**

**Dr Ashley Elliott**

**MUSCULOSKELETAL ULTRASOUND TRAINING LOGBOOK**

**Name:­** _____________________________________________

**GMC Number:** _______________________________________

**CONTENTS TABLE:**

|  | Page Number |
| --- | --- |
| **Recommended timeframe** | **6** |
| **Curriculum** | **7** |
| **Training plan** | **9** |
| **Courses attended (+ certificates)** | **10** |
| **Structured ultrasound teaching attended** | **11** |
| **Logbook** | **12** |
| Shoulder | 12 |
| Elbow | 14 |
| Wrist | 16 |
| Hand | 18 |
| Hip | 20 |
| Knee | 22 |
| Ankle | 24 |
| Foot | 26 |
| Temporal Arteries | 28 |
| Miscellaneous | 29 |
| **Assessments (CBDs/DOPS)** | **30** |
| **EFSUMB Level 1 Competency Sheet** | **32** |
| **Let’s play Bingo!** | **36** |
| **References** | **37** |

**RECOMMENDED TIMEFRAME:**

ST4-5

Attend organised teaching sessions of normal joint scans

Sign up for the EULAR Online US course + sit the exam

Consider attending basic US course

ST6

Attend basic US Course (if not attended in previous years)

Attend intermediate US course

Collect images for NORMAL joint scans

Begin collecting images for logbook

ST7

Aim to sit the EFSUMB Level 1 Competency exam by the end of the training year

Ensure logbook completed (to include 300 joint examinations)

Consider attending Advanced US course

**CURRICULUM:**

The European Federation of Societies for Ultrasound in Medicine and Biology (EFSUMB) governs training and practice standards in Europe and has published Mimimum Training Requirements for Rheumatologists performing Musculoskeletal Ultrasound (www.efsumb.org) in conjunction with the EULAR which is the representative body for all rheumatology societies in Europe. These recommendations establish the theoretical and practical curriculum for training and achieving competency in POCUS in rheumatology. On obtaining competency recorded in this logbook an EFSUMB Level 1 certificate may be awarded by a Level 3 certificate holder. Table 1 lists the theoretical competency requirements. These are met by completion of the EULAR online course which is subject to an examination and certificate and/or by satisfactory attendance at accredited basic and intermediate ultrasound course as assessed by the fellow’s supervisor.

**Table 1: Level 1 EFSUMB competency requirements for rheumatologists who perform musculoskeletal ultrasound (MSUS)**

**Level 1: Theoretical Module**

Attendance in a basic and intermediate MSUS course, each of at least 18 hours and at least 50% of supervised practical sessions during the last 3 years (for beginners) or at least 2 update MSUS courses during the last 5 years for rheumatologists with experience in performing MSUS. The required MSUS courses should be endorsed by national or international scientific societies. The content of these courses should include the following topics:

- Application, indications and limitations of MSUS in rheumatology.
- Ultrasound physics and technology
- Sonographic pattern of the different musculoskeletal tissues
- MSUS artefacts and pitfalls
- Standard sonographic scans of the shoulder, elbow, wrist and hand, hip, knee, ankle and foot
- Holding the probe and optimizing the grey-scale settings of the system
- Image documentation
- Reporting ultrasound findings and diagnosis
- Colour and power Doppler physics and technology
- Application, indications and limitations of colour and power Doppler in rheumatology
- Use and optimization of colour and power Doppler settings
- Colour and power Doppler artefacts
- Use of colour and power Doppler to detect synovial and entheseal inflammation
- Assessment of synovial, tenosynovial and entheseal inflammatory activity with B-mode and Doppler
- Assessment of structural joint damage (bone, cartilage, tendons, ligaments)
- Sonographic-guided periarticular and intra-articular injections
- Ultrasound detection of the following pathologies: joint synovitis (i.e. synovial hypertrophy and joint effusion), tenosynovitis, bursitis, enthesopathy, paratenonitis, tendinosis, complete tendon tear, partial tendon tear, tendon impingement, bony cortex abnormalities (i.e. bone erosions, enthesophytes, osteophytes), ganglia and cysts, articular cartilage lesions, peri- and intra-articular microcrystal deposit, and MSUS calcification

Practical Training must be undertaken at an accredited centre under the supervision of EFSUMB level 2 or 3 certified trainers with appropriate ultrasound imaging equipment and dedicated ultrasound clinics.

A logbook must be completed containing the minimum number of cases and with satisfactory completion of Directly Observed Procedures (DOPs) and Case Based Discussion (CBD) as assessed by a level 2 or 3 certified trainer. A minimum of 2 Level 2/3 trainers will have evaluated each fellow for certification. This may require an external assessor if a training centre only has one Level 2/3 trainer.

**Table 2: Level 1: Practical Training**

Level 1 competence requires the following practical competencies:

• To obtain level 1 status it is recommended that the trainee should perform a minimum of 300 joint region examinations (different joints) under supervision (as listed in the competency assessment sheet) within 1 year.

• Examinations should encompass the full range of conditions listed in the competency assessment sheet - level 1.

• A log book (or an illustrated log book) should be kept by the trainee, listing the number and type of examinations and reporting and documenting the ultrasound findings. Supervision of half of the 300 examinations can be achieved with approval of examinations in an illustrated log book.

• The trainee should be supervised by a level 2 or 3 practitioner. During the course of training a competency assessment sheet should be completed and signed by the supervisor, as this will determine in which area(s) the trainee can practise independently. To maintain level 1 status the practitioner is expected to perform at least 300 examinations each year.

**Level 1: Competencies to be acquired**

At the end of training the trainee should be able to:

• Perform common musculoskeletal ultrasound examinations (shoulder, elbow, wrist/hand, hip, knee, ankle/foot and muscles) systematically, accurately, safely and with proper report and documentation.

• Differentiate normal from common pathological findings listed in the competency assessment sheet - level 1.

• Recognize when referral for an additional opinion is indicated (e.g. any diagnostic doubt), both from more experienced colleagues or by use of other imaging techniques.

• Diagnose common abnormal ultrasound findings as listed in the competency assessment sheet - level 1.

• To perform basic US-guided interventions

**TRAINING PLAN:**

|  | Current status | Plan | Consultant sign off |
| --- | --- | --- | --- |
| EULAR online course |  |  |  |
| Basic course |  |  |  |
| Intermediate course |  |  |  |
| Advanced course |  |  |  |
| Other courses |  |  |  |
| Logbook cases |  |  |  |
| DOPs |  |  |  |
| Competency exam |  |  |  |
| Research* |  |  |  |
| Audit* |  |  |  |

***Include audit or research abstracts in the logbook**

**COURSES (insert certificates):**

1. **EULAR Online Introductory Ultrasound Course**
2. **Basic course**
3. **Intermediate course**
4. **Advanced course**
5. **Other courses**

| **Course** | **Date Completed** | **Supervisor Sign-off** |
| --- | --- | --- |
| **EULAR Online Introductory Ultrasound Course** |  |  |
| **Basic Course** |  |  |
| **Intermediate Course** |  |  |
| **Advanced Course** |  |  |
| **Other** |  |  |

**STRUCTURED ULTRASOUND TEACHING:**

| **Teaching** | **Location** | **Date** |
| --- | --- | --- |
| Musgrave Park Hospital in house training **SHOULDER** (normal) |  |  |
| Musgrave Park Hospital in house training **ELBOW** (normal) |  |  |
| Musgrave Park Hospital in house training **HAND and WRIST** (normal) |  |  |
| Musgrave Park Hospital in house training **HIP** (normal) |  |  |
| Musgrave Park Hospital in house training **KNEE** (normal) |  |  |
| Musgrave Park Hospital in house training **ANKLE and FOOT** (normal) |  |  |
| Musgrave Park Hospital in house training **VASCULAR SCANNING** (normal + pathology) |  |  |
| Northern Ireland Ultrasound Teaching Day |  |  |
|  |  |  |
|  |  |  |
|  |  |  |

**LOGBOOK:**

*****NOTE:** 300 scans in total are required. There should be minimum of 25 scans per joint.***

1. **Shoulder**

| **Date** | **Patient ID** |  | **Clinic** |
| --- | --- | --- | --- |
|  | **EXAMPLE** | R shoulder subscapularis tendon calcification |  |
|  |  |  |  |

2. **Elbow**

| **Date** | **Patient ID** |  | **Clinic** |
| --- | --- | --- | --- |
|  | **EXAMPLE** | Left elbow joint effusion |  |
|  |  |  |  |

3. **Wrist**

| **Date** | **Patient ID** |  | **Clinic** |
| --- | --- | --- | --- |
|  | **EXAMPLE** | Right wrist dorsal median longitudinal scan – *joint effusion; grade 1 power Doppler positive |  |
|  |  |  |  |

4. **Hand**

| **Date** | **Patient ID** |  | **Clinic** |
| --- | --- | --- | --- |
|  | **EXAMPLE** | Dorsal longitudinal R 2^nd^ DIP scan - Osteophyte |  |
|  |  |  |  |

5. **Hip**

| **Date** | **Patient ID** |  | **Clinic** |
| --- | --- | --- | --- |
|  | **EXAMPLE** | Right hip effusion |  |
|  |  |  |  |

6. **Knee**

| **Date** | **Patient ID** |  | **Clinic** |
| --- | --- | --- | --- |
|  | **EXAMPLE** | Patellar tendon tendinopathy and infrapatellar bursitis |  |
|  |  |  |  |

7. **Ankle**

| **Date** | **Patient ID** |  | **Clinic** |
| --- | --- | --- | --- |
|  | **EXAMPLE** |   Longitudinal right tibio-talar scan - effusion |  |
|  |  |  |  |

8. **Foot**

| **Date** | **Patient ID** |  | **Clinic** |
| --- | --- | --- | --- |
|  | **EXAMPLE** | Longitudinal 1^st^ left MTPJ scan – double contour sign |  |
|  |  |  |  |

9. **Temporal Arteries**

| **Date** | **Patient ID** |  | **Clinic** |
| --- | --- | --- | --- |
|  | **EXAMPLE** | Normal |  |
|  |  |  |  |

10. **Miscellaneous (e.g. salivary gland, lung, skin)**

| **Date** | **Patient ID** |  | **Clinic** |
| --- | --- | --- | --- |
|  |  |  |  |
|  |  |  |  |
|  |  |  |  |

**ASSESSMENTS:**

1. **Directly Observed Procedures (DOPS)**

**DOPS -Perform standard EULAR ultrasound scan/injection**

| Date: | ID: |
| --- | --- |
| Procedure: (Shoulder, Elbow, Wrist, Hand, Hip, Knee, Ankle, Foot, TAUS, Other_______) | |
| Details of Procedure (Write report of scan below) | |
| Examiner Score:  0. Obtains wrong view or does not obtain image   1. Obtains correct view, some optimisation of image and identifies a few of the structures 2. Obtains correct view, competent probe handling and panning, adequate optimisation of image and identifies most of structures 3. As per 3, excellent optimisation of image (correct probe and frequency selection, focussing, depth setting), competent optimisation of power Doppler if relevant and identifies all of anatomical structures 4. As per 3 and correctly identifies the pathology (if relevant, may be normal scan) | |
| Examiner comment | |
| Consultant sign off | |

2. **Case Based Discussion (CBD)**

Instructions

- A CBD is intended for structured review of case experience between Trainee and Trainer. It is a critical appraisal of the reasoning and judgements made, and of the management of the case. This form should be completed jointly with your Trainer as part of the CBD

**New Case Based Discussion: Perform standard EULAR ultrasound scan / injection**

| Date: | ID: |
| --- | --- |
| Procedure: (Shoulder, Elbow, Wrist, Hand, Hip, Knee, Ankle, Foot, TAUS, Other_______) | |
| Brief Description of Case (Write report and reflection of scan below ) | |
| Examiner Score of imaging:   0. Obtains wrong view or does not obtain image 1. Obtains correct view, some optimisation of image and identifies a few of the structures 2. Obtains correct view, competent probe handling and panning, adequate optimisation of image and identifies most of structures 3. As per 3, excellent optimisation of image (correct probe and frequency selection, focussing, depth setting), competent optimisation of power Doppler if relevant and identifies all of anatomical structures 4. As per 3 and correctly identifies the pathology (if relevant, may be normal scan) | |
| Examiner comment on reflection: | |
| Consultant sign off | |

**EFSUMB competency sheet for rheumatologists:**

Core Knowledge Base – Level 1

Trainee________________________________

**Musculoskeletal Competency Sheet**

| **Normal Joints** | **Date** | **Trainer signature** |
| --- | --- | --- |
| Shoulder |  |  |
| Elbow |  |  |
| Wrist |  |  |
| Hip |  |  |
| Knee |  |  |
| Ankle |  |  |
| Foot |  |  |

| **Pathology Shoulder** | **Date** | **Trainer signature** |
| --- | --- | --- |
| Glenohumeral synovitis / effusion (B-mode and Doppler) |  |  |
| Acromioclavicular abnormalities |  |  |
| Biceps sheath effusion |  |  |
| Biceps tendinopathy/rupture |  |  |
| Rotator cuff calcification |  |  |
| Rotator cuff full-thickness tear |  |  |
| Rotator cuff partial-thickness tear |  |  |
| Supraspinatus impingement |  |  |
| Subacromial – subdeltoid bursitis |  |  |
| Bone Erosions |  |  |
| Osteophytes |  |  |
| Other pathologies |  |  |
| US- guided injections |  |  |

| **Pathology Elbow** | **Date** | **Trainer signature** |
| --- | --- | --- |
| Synovitis (B-mode and Doppler) |  |  |
| Extensor enthesopathy (B-mode and Doppler) |  |  |
| Flexor enthesopathy (B-mode and Doppler) |  |  |
| Triceps enthesopathy (B-mode and Doppler) |  |  |
| Extensor tendinopathy/ rupture |  |  |
| Bone erosions, enthesophytes, osteophytes |  |  |
| Other pathologies |  |  |
| US-guided injections |  |  |

| **Pathology Wrist and Hand** | **Date** | **Trainer signature** |
| --- | --- | --- |
| Wrist Synovitis (B-mode and Doppler) |  |  |
| Wrist tenosynovitis (B-mode and Doppler) |  |  |
| Metacarpophalangeal synovitis (B-mode and Doppler) |  |  |
| Interphalangeal synovitis (B-mode and Doppler) |  |  |
| Finger flexor tenosynovitis (B-mode and Doppler) |  |  |
| Finger extensor paratenonitis (B-mode and Doppler) |  |  |
| Wrist ganglia and cysts |  |  |
| Bone erosions |  |  |
| Osteophytes |  |  |
| Other pathologies |  |  |
| US-guided injections |  |  |

| **Pathology Hip** | **Date** | **Trainer signature** |
| --- | --- | --- |
| Synovitis (B-mode and Doppler) |  |  |
| Iliopsoas bursitis |  |  |
| Gluteus enthesopathy/tendinopathy/rupture |  |  |
| Trochanteric bursitis |  |  |
| Osteophytes |  |  |
| Other pathologies |  |  |
| US-guided injections |  |  |

| **Pathology Knee** | **Date** | **Trainer signature** |
| --- | --- | --- |
| Synovitis (B-mode and Doppler) |  |  |
| Quadriceps enthesopathy (B-mode and Doppler) |  |  |
| Patellar enthesopathy (B-mode and Doppler) |  |  |
| Patellar tendinopathy/rupture |  |  |
| Tendon/ligament/cartilage microcrystal deposit |  |  |
| Prepatellar /infrapatellar bursitis |  |  |
| Femoral cartilage bursitis |  |  |
| Baker’s cyst |  |  |
| Enthesophytes |  |  |
| Osteophytes |  |  |
| Other pathologies |  |  |
| US-guided injections |  |  |

| **Pathology Ankle and Foot** | **Date** | **Trainer signature** |
| --- | --- | --- |
| Tibiotalar/subtalar/talar synovitis (B-mode and Doppler) |  |  |
| Metatarsophalangeal synovitis (B-mode and Doppler) |  |  |
| Interphalangeal synovitis (B-mode and Doppler) |  |  |
| Ankle tenosynovitis (B-mode and Doppler) |  |  |
| Foot tenosynovitis (B-mode and Doppler) |  |  |
| Tarsal ganglia |  |  |
| Achilles enthesopathy (B-mode and Doppler) |  |  |
| Achilles tendinopathy/rupture |  |  |
| Retrocalcaneal bursitis |  |  |
| Other bursitis |  |  |
| Plantar fasciitis |  |  |
| Bone erosions |  |  |
| Enthesophytes, osteophytes |  |  |
| Cartilage microcrystal deposit |  |  |
| Other pathologies |  |  |
| US-guided injections |  |  |

**TAUS Competency Sheet**

| **Normal Scans** | **Date** | **Trainer Signature** |
| --- | --- | --- |
| Common Temporal Artery |  |  |
| Frontal Temporal Artery |  |  |
| Parietal Temporal Artery |  |  |
| Axillary Artery |  |  |
| **Pathology Common Temporal Artery** |  |  |
| IMT thickness measurement |  |  |
| Halo sign |  |  |
| Non-compressibility |  |  |
| Occlusion |  |  |
| Stenosis |  |  |
| **Pathology Frontal Temporal Artery** |  |  |
| IMT thickness measurement |  |  |
| Halo sign |  |  |
| Non-compressibility |  |  |
| Occlusion |  |  |
| Stenosis |  |  |
| **Pathology Parietal Temporal Artery** |  |  |
| IMT thickness measurement |  |  |
| Halo sign |  |  |
| Non-compressibility |  |  |
| Occlusion |  |  |
| Stenosis |  |  |
| **Pathology Axillary Artery** |  |  |
| IMT thickness measurement |  |  |
| Halo sign |  |  |
| Occlusion |  |  |
| Stenosis |  |  |
| Atherosclerosis |  |  |
| Slope sign |  |  |

**LET’S PLAY BINGO!**

**BINGO RULES:**

1. The aim of the game is to motivate trainees and help to guide and prioritise MSK US learning and logbook completion.
2. Topics for the card were selected from EFSUMB Level 1 MSK US Curriculum.
3. ‘BINGO’ game **starts in February 2026 and will continue until July 2026**.
4. ‘BINGO’ can be achieved by having **five** consecutive squares (either horizontally, vertically or diagonally) signed off by a supervising consultant with accompanying images included in the logbook.
5. The first trainee to report ‘BINGO’ from February 2026 to July 2026 (following satisfactory review of images and sign-off) will be given a certificate and a small prize.

**REFERENCES:**

1. Atchia I, Brown AK, Chitale S, Ciechomska A, Estrach C, Karim Z, Wakefield RJ; British Society for Rheumatology Ultrasound Special Interest Group (BSRUSSIG). Recommendations for rheumatology ultrasound training and practice in the UK. Rheumatology (Oxford). 2021 Jun 18;60(6):2647-2652. doi: 10.1093/rheumatology/keaa656. PMID: 33167033.
2. A. Taggart, E. Filippucci, G. Wright, A. Bell, A. Cairns, G. Meenagh, A. Pendleton, M. Rooney, S. Wright, A. Grey, W. Grassi, Musculoskeletal ultrasound training in rheumatology: the Belfast experience, Rheumatology, Volume 45, Issue 1, January 2006, Pages 102–105, https://doi.org/10.1093/rheumatology/kei162
3. Terslev L, Hammer HB, Torp-Pedersen S, Szkudlarek M, Iagnocco A, D'Agostino MA, Schmidt WA, Uson J, Bruyn GA, Filippucci E, Möller I, Balint P, Wakefield R, Naredo E. EFSUMB minimum training requirements for rheumatologists performing musculoskeletal ultrasound. Ultraschall Med. 2013 Oct;34(5):475-7. doi: 10.1055/s-0033-1335143. Epub 2013 May 21. PMID: 23696065.
4. Pfeil A, Krusche M, Vossen D, Berliner MN, Keyßer G, Krause A, Lorenz HM, Manger B, Schuch F, Specker C, Wollenhaupt J, Baraliakos X, Fleck M, Proft F. Model curriculum of the German society for Rheumatology for advanced training in the discipline internal medicine and rheumatology. English version. Z Rheumatol. 2021 Dec;80(Suppl 2):64-67. doi: 10.1007/s00393-021-01080-6. Epub 2021 Oct 4. PMID: 34605981; PMCID: PMC8752525.
5. Gillispie-Taylor M. Bingo! Gamifying Pediatric Rheumatology Education One Square at a Time. Perspectives on Medical Education. 2025; 14(1): 641–646. DOI: https://doi. org/10.5334/pme.1749
